# Soccer in the time of COVID-19: 1 year report from an Italian top league club, March 2020–February 2021

**DOI:** 10.1017/S0950268821002065

**Published:** 2021-09-08

**Authors:** Michele Spinicci, Luca Pengue, Dario Bartolozzi, Massimo Quercioli, Francesco Epifani, Simona Pollini, Lorenzo Zammarchi, Gian Maria Rossolini, Alessandro Bartoloni

**Affiliations:** 1 Department of Experimental and Clinical Medicine, University of Florence, Florence, Italy; 2 Infectious and Tropical Diseases Unit, Careggi University Hospital, Florence, Italy; 3 Sports Medicine Unit, Fondazione Policlinico Universitario Agostino Gemelli IRCCS, Rome, Italy; 4 Synlab Med, Florence, Italy; 5 Microbiology and Virology Unit, Careggi University Hospital, Florence, Italy

**Keywords:** COVID-19, football, outbreaks, SARS-CoV-2, sport

## Abstract

We report the events of an Italian top league soccer club that took place in 1 year (from March 2020 to February 2021) at the time of coronavirus disease 2019 (COVID-19) pandemic. In early March 2020, just before sport competitions were called off due to the national lockdown in Italy, the team, which included 27 players and 26 staff at the time, faced a COVID-19 outbreak, with 16 confirmed and seven probable cases, including three staff members who had to be hospitalised. In May 2020, at the resumption of the training sessions, a high prevalence of anti-severe acute respiratory syndrome coronavirus 2 (SARS-CoV-2) immunoglobulin G positivity (35/53, 66%) was detected among the members of the group. In the following months, sport activities were organised behind closed doors with stringent risk mitigation procedures in place. As of February 2021, only two new cases of SARS-CoV-2 infection were detected within the group, against more than 3500 nasopharyngeal swabs and 1000 serological tests.

## Introduction

Since the beginning of 2020, coronavirus disease 2019 (COVID-19), caused by severe acute respiratory syndrome coronavirus 2 (SARS-CoV-2), emerged as a major public health concern worldwide, with a disrupting impact on day-to-day activities [1].

In the first few months of the pandemic, all sport competitions and team sport-training sessions were called off in most of the countries worldwide. Major international sport events, scheduled for the summer 2020, such as the European Football Championship and the Olympic Games in Tokyo, were postponed for a year. At local and international levels, a lively debate arose about whether, how and when pending sports competitions should be re-started or, otherwise, be cancelled [2–4]. Then, from May 2020, main soccer competitions, such as UEFA European Football Championship, UEFA Champions League and almost all major national leagues, were conducted behind closed doors with stringent risk mitigation procedures in place.

The early experience from restart of professional soccer league in Germany (study period: May–July 2020) and Qatar (study period: June–September 2020) suggests that both training and official matches could be carried out safely during the pandemic, even when the burden of viral transmission is high in the general population [5, 6]. Beyond soccer, also data from the US National Football League (August–November 2020) indicated that a relative safety of professional sports team competition could be achieved [7]. Strict hygiene measures and regular testing by reverse-transcriptase polymerase chain reaction (RT-PCR) were the key factors for these results. Moreover, studies focused on infection-relevant contacts during soccer and rugby matches, in which COVID-19-positive players were found to have participated, documented a very low risk of transmission ‘on the pitch’, among teammates, opponent players and also referees [8, 9].

In this paper, we describe the events of an Italian top league soccer club that took place in 1 year (from March 2020 to February 2021), focusing on the dynamics of SARS-CoV-2 transmission within the group and the impact of mitigation strategies implemented by the Italian Football Federation (Italian: Federazione Italiana Giuoco Calcio; FIGC).

## Epidemiology

In Italy, where the first confirmed autochthonous cases date back to 21 February 2020, a national quarantine, aimed to tackle the spread of the disease by a stay-home mandate and closure of all non-essential productive activities, was imposed from 9 March 2020 [10]. The top Italian soccer league, known as Serie A, was similarly suspended, with the last matches played on 8 March 2020.

In March 2020, in the early phase of the pandemic, an Italian top league soccer team, which included 27 players and 26 staff at that time, faced a COVID-19 outbreak. In total, 16 people were diagnosed with the disease (five players and 11 staff members) by RT-PCR on nasopharyngeal swab (NPS), 5 to 7 days after their last meeting, which occurred just before the national lockdown was clamped. In addition, seven players and one staff member experienced mild symptoms of COVID-19 but RT-PCR on NPS was not obtained. All but three developed a mild form of the disease without signs of lower respiratory tract infection or pneumonia. Three staff members (44-year-old, 39-year-old and 56-year-old) developed moderate to severe symptoms and were hospitalised.

Among close contacts of the players and staff, at least five cases were subsequently reported, representing possible secondary cases due to in-family transmission. Four of them were hospitalised (age range: 76–90), including two cases that required admission to an intensive care unit. One of them died (a 90-year-old woman).

On 18 May 2020, as soon as the national lockdown was removed, team sport-training sessions restarted under a strict protocol for COVID-19 surveillance issued by FIGC. All team players and staff members were screened 72–96 h before the first training session by both SARS-CoV-2 RT-PCR on NPS and immunoglobulin G (IgG)/IgM serological testing, and then repeated every 4 and 14 days, respectively. Mitigation strategies included physical distancing (>2 m) and wearing masks during practice sessions (only for staff members), meetings, medical treatments and rehabilitation sessions, inside the dressing room, and meal area. Indoor activities, such as gym sessions were kept to an absolute minimum. Prevention measures during indoor trainings were guaranteed by alternating small groups of athletes, and by placing gym equipment at least 2 m apart, in a well-ventilated room with forced ventilation, and ensuring deep sanitation of the instruments. If a positive case was detected, the entire group must be placed in a ‘bubble’, i.e. confined for 2 weeks in a dedicated facility identified by the club, and tested by NPS every 48 h and serology at the baseline and after 10 days. During the quarantine period, contact with people outside the team group, including relatives, was forbidden, but training and official matches were permitted [11].

The first screening performed in early May 2020, before the activity resumed, showed a high prevalence of IgG positivity (35/53, 66%, including 18/27, 67% players and 17/26, 65% staff members). At this time, three players and three staff members had detectable viral RNA by RT-PCR on NPS: among them, five had previous positive RT-PCR and one reported mild symptoms, but did not seek RT-PCR testing in March.

During the following period until the end of the season on 2 August 2020, the entire group performed RT-PCR and serological tests every 4 and 14 days, respectively, as per the FIGC protocol, and no new case of infection was detected.

After a 3-week break, on 25 August 2020, the team members were called to restart training, in view of the new Serie A season. At the baseline screening, performed before the first meeting, a new case of asymptomatic COVID-19 was detected by RT-PCR on NPS, among the 54 members of the group (28 players, including 11 new ones with respect to the previous season, and 26 staff members). The player, who had tested negative to all the previous serological and molecular testing, was confined from the rest of the group, until achieving SARS-CoV-2 clearance by RT-PCR on NPS. Molecular and serological assessment continued with the same frequency of the previous season (every 4 and 14 days, respectively) until 28 September 2020, when the FIGC protocol was revised and molecular testing was required only within 48 h before the soccer match, while serological monitoring was maintained every 14 days. A single case of asymptomatic positivity was detected in November 2020, in a player transferred a month before from another team. The group was confined in a ‘bubble’ according to the FIGC protocol for 14 days, and no secondary cases emerged among teammates.

As of 28 February 2021, no further cases of COVID-19 were detected within the group (now consisting of 25 players and 26 staff members after the winter transfer window). To date, more than 3500 RT-PCR tests and 1000 serological tests were performed to comply with the FIGC surveillance protocol, since the resumption of the sport activities in May 2020.

The study was performed in accordance with the ethical principles of the Declaration of Helsinki and with the International Conference on Harmonization Good Clinical Practice guidelines. Participants' written consent was obtained. All tests performed were part of the medical care, according to the FIGC protocol.

## Discussion

High risk of COVID-19 transmission has been previously reported in relation to specific sport activities, such as hockey, dance and squash [12–14]. Several factors can contribute to increase the risk of respiratory virus transmission within a sports group. Contact sports do not allow compliance with the normal preventive rules recommended by World Health Organization [15]. Actually, during a soccer match, players cannot voluntarily avoid the contact with other players, and intense physical exercise can result in incremented release of isolated droplets [16]. However, the total exposure of a soccer player within a potentially risky zone of 1.5 m from a given opponent player was estimated to be limited in time (34–115 s/h), during recreational small-sided football games [17]. To a certain extent, even the contact with ball and other equipment can lead to infection, although there is uncertainty about the role of fomites in COVID-19 transmission [18]. Notably, a rapid decay in transmissible SARS-CoV-2 virus on several types of sports equipment has been documented, with a recoverable viral load on all materials less than 1% of the original inoculum after 1 min [19]. Then, outside the pitch, athletes and staff share for long time closely confined spaces such as dressing rooms, where moist, warm atmosphere, could facilitate contagion. Finally, professional soccer players are generally young and healthy individuals without chronic conditions, therefore having higher probability to develop a-/pauci-symptomatic infections [20]. Asymptomatic cases are at risk to remain undiagnosed and confinement measures could be delayed. On the contrary, players and staff members may have contact with older subjects – at higher risk to develop more severe forms of COVID-19 – as possibly occurred with four elderly people in the reported cluster.

Since social distancing and other prevention measures may not be possible in all circumstances, application of strict mitigation strategies combined with regular testing is critical to overcome the pandemic challenge [21]. To date, available data from leading professional soccer and football leagues are reassuring, since competitions have been successfully restarted under the pandemic situation, without the evidence of meaningful COVID-19 transmission [5–7]. Also, the FIGC protocol proved to be apparently effective at reducing the SARS-CoV-2 transmission within professional clubs. In our case, the infection was caused due to an outbreak that occurred in the very early phase of the pandemic, before introduction of specific mitigation strategies for sport activities. Subsequently, only two cases of COVID-19, possibly caused due to community exposure, were diagnosed since the restart of the training sessions in May 2020, without the evidence of secondary cases. With preventive measures in place, the risk of COVID-19 transmission for athletes and staff was low, compared to the infection rate in the community, which reached the peak in November 2020 in Italy [22]. By contrast, association between new weekly COVID-19 cases within professional rugby and new weekly COVID-19 cases within the community was observed in England [23]. In our case, a contributing role could be played by the high frequency of persistent immunity against COVID-19 among the members of the group, being an immunological signature detected in 24/54 (44%) of group members at the beginning of the 2020–21 season in August 2020, 5 months after the potential exposure [24].

The same strategies are not sustainable for amateur sports and recreational athletes. However, there is much supporting evidence that the risk of COVID-19 transmission during outdoor training and games has been likely overestimated, and several warnings and recommendations against playing soccer, as well as other contact sports, may be revised. Larger studies on soccer-specific contacts, which take into account contact tracing frameworks to identify high risk interactions, may help to substantiate these findings [25]. Health and sports authorities are called to provide strategies that harmonise the importance to maintain sport activities at all levels, with the safety of athletes, staff and their relatives.

Last but not least, maximum caution must be applied when athletes who experienced symptoms of COVID-19 resume sports activities, since long-term outcomes for patients who contracted the disease are still be determined [26, 27]. Therefore, specific protocols to check for cardiological, pulmonary and other systemic sequelae of COVID-19 in the athletes are warranted. Nonetheless, a recent study performing an extensive cardio-pulmonary screening in athletes with previous asymptomatic or mild SARS-CoV-2 infection did not identify any pathologic finding [28].

## Data Availability

The data that support the findings of this study are available on request from the corresponding author. The data are not publicly available due to restriction regarding participants' privacy.
